# Developing a Novel Adaptive Double Deep Q-Learning-Based Routing Strategy for IoT-Based Wireless Sensor Network with Federated Learning

**DOI:** 10.3390/s25103084

**Published:** 2025-05-13

**Authors:** Nalini Manogaran, Mercy Theresa Michael Raphael, Rajalakshmi Raja, Aarav Kannan Jayakumar, Malarvizhi Nandagopal, Balamurugan Balusamy, George Ghinea

**Affiliations:** 1 Department of Computer Science and Business Systems, S.A. Engineering College (Autonomous), Chennai 600077, Tamil Nadu, India; drnalini@saec.ac.in; 2 Department of Data Science and Business Systems, School of Computing, College of Engineering and Technology, SRM Institute of Science and Technology, Chennai 603203, Tamil Nadu, India; mercythm@srmist.edu.in; 3 Department of Computer Science and Engineering, Sathyabama Institute of Science and Technology, Chennai 600119, Tamil Nadu, India; rajalakshmi.cse@sathyabama.ac.in; 4 Department of BioMedical Engineering, SRM Institute of Science and Technology, Chennai 600089, Tamil Nadu, India; ak9528@srmist.edu.in; 5 Department of Computer Science and Engineering, School of Computing, Vel Tech Rangarajan Dr. Sagunthala R&D Institute of Science and Technology, Chennai 600062, Tamil Nadu, India; drnmalarvizhi@veltech.edu.in; 6 Associate Dean Academics, Office of Dean of Academics, Shiv Nadar University, Delhi-National Capital Region (NCR), Dadri 201314, Uttar Pradesh, India; balamurugan.balusamy@snu.edu.in; 7 Department of Computer Science, College of Engineering Design and Physical Sciences, Brunel University London, Uxbridge UB8 3PH, UK

**Keywords:** Internet of Things (IoT), wireless sensor network (WSN), smart data routing, federated learning, deep earning

## Abstract

The working of the Internet of Things (IoT) ecosystem indeed depends extensively on the mechanisms of real-time data collection, sharing, and automatic operation. Among these fundamentals, wireless sensor networks (WSNs) are important for maintaining a countenance with their many distributed Sensor Nodes (SNs), which can sense and transmit environmental data wirelessly. Because WSNs possess advantages for remote data collection, they are severely hampered by constraints imposed by the limited energy capacity of SNs; hence, energy-efficient routing is a pertinent challenge. Therefore, in the case of clustering and routing mechanisms, these two play important roles where clustering is performed to reduce energy consumption and prolong the lifetime of the network, while routing refers to the actual paths for transmission of data. Addressing the limitations witnessed in the conventional IoT-based routing of data, this proposal presents an FL-oriented framework that presents a new energy-efficient routing scheme. Such routing is facilitated by the ADDQL model, which creates smart high-speed routing across changing scenarios in WSNs. The proposed ADDQL-IRHO model has been compared to other existing state-of-the-art algorithms according to multiple performance metrics such as energy consumption, communication delay, temporal complexity, data sum rate, message overhead, and scalability, with extensive experimental evaluation reporting superior performance. This also substantiates the applicability and competitiveness of the framework in variable-serviced IoT-oriented WSNs for next-gen intelligent routing solutions.

## 1. Introduction

WSNs have developed as a primary technology for sending and garnering data in distinct application sectors because of the IoT device’s proliferation [1]. Because of the frequent relocation of SNs and their dynamic nature, the IoT-allows to WSNs encounter specific complexities concerning data routing. IoT-aided WSNs should route information across different access points while handling effective transmission and preserving energy for optimal functionality [2]. The conventional routing approaches may face complexities in resolving these limitations efficiently, resulting in less network lifespan, enhanced latency, and the selection of packet suboptimal nodes [3]. Often, the routing of data in IoT-based WSNs faces multiple complexities. The SN’s frequent relocation presents extra complexities and demands adaptive routing mechanisms for handling the dynamic network topologies efficiently. The WSN’s resource-constrained character which contains the constrained computing capabilities and energy availability, demands energy-efficient routing approaches for enhancing the lifetime of the network [4]. The routing protocol’s scalability is significant for allocating the huge IoT system’s deployment and a vast amount of data produced [5,6]. The heterogeneous and dynamic nature of IoT-based WSNs demands the routing mechanisms that allocate distinct node types, such as non-mobile as well as mobile models while handling efficient communication and reducing delay.

In real-time, the IoT-enabled devices can send and garner data, allowing for response and validation of dynamic conditions in the system [7]. This is developed to be energy-effective, which is vital for enhancing the SN’s operational lifespan in WSNs [8]. This efficacy supports optimizing energy usage during the transmission of data as well as acquisition. The IoT-aided devices can adapt to the dynamic environment, including distinct network conditions or node relocations, guaranteeing that data acquisition remains efficient even in varying situations [9]. By automating data acquisition and minimizing the requirement for manual analysis, IoT devices can minimize the operational expenses related to data management and monitoring. This includes methods such as transmission scheduling and path selection to guarantee accurate and effective data transmission among nodes [10,11]. The experiment of the routing strategy in WSN is highly important for enhancing the functionality of the network, increasing the lifetime of the network, offering better data transmission, and helping the applications in real-time [12]. By employing efficient routing optimization approaches, highly effective, intelligent, and reliable WSN devices can be obtained [13,14]. The WSN routing optimization plays a significant part in IoT, as it can enhance the efficiency of the energy utilization, and network functionality, extend the lifespan of the network, offer better transmission, and help the real-time applications for diverse sectors. Hence, the routing optimization in WSN is highly important in enhancing the application as well as the application of animal networking [15].

The conventional routing approaches for IoT-based WSNs often fail to resolve these problems sufficiently [16]. These models have poor adaptability for relocating the node, energy efficiency optimization, and also scalability [17,18,19]. Moreover, including Deep Reinforcement Learning (DRL) models in routing models for WSNs remains undiscovered [20]. The problem lies in the implementation of a smart data routing mechanism on the basis of federated DRL that considers distinct attributes related to the routing [21], and this contains the data sum rate, message overhead, time complexity, energy efficiency, communication delay, and scalability while managing the optimal network functionality and also node relocations. Typically, SNs have constrained energy resources [22]. The conventional routing approaches may not efficiently optimize energy usage, resulting in minimized network lifespan and premature node failure. To overcome these challenges, an efficient routing model is implemented in this proposed model.

The implemented data routing system for IoT-aided WSN contains the contributions below.

- To perform data routing for IoT-aided WSN utilizing FL and the Q-learning-based approach. This data routing process allows the optimal path selection for transmitting the data packets from source to destination without any loss of packets and interruptions. The data routing process supports minimizing energy usage by choosing the optimal paths, thus extending the life span of the network. Moreover, this process improves the efficiency, reliability, and performance of the network by carefully selecting the best paths for data transmission in IoT-based WSNs.
- To design IRHO for fine tuning the parameters. The IRHO is developed by enhancing the exploitation stage of the traditional Hippopotamus Optimization Algorithm (HOA) with the support of an iteration-based random factor. This enhanced exploitation stage increases algorithm convergence and helps explore suitable solutions for the optimization problems. Moreover, by upgrading the conventional HOA, the designed IRHO mitigates the concern of premature convergence and also avoids high computations. The IRHO supports DDQL in choosing its parameters optimally during the data routing and decision-making operations thus helping to enhance the Packet Delivery Ratio (PDR), and minimize the delay and energy consumption.
- To present a novel FL-based ADDQL for performing the data routing and decision-making operations in IoT-based WSN. FL enables the distributed SNs in a WSN to collaborate and learn without transmitting original data to the intermediate server. The designed ADDQL learns the routing decisions and optimal policies on the basis of environmental feedback, thus optimizing the network performance. Here, the DDQL parameters are optimally selected by the IRHO. Thus, the integration of FL and ADDQL in data routing and decision-making operations in IoT-based WSN leads to enhanced efficiency, energy savings, adaptability, robustness, privacy, and scalability. These merits make the approach relatively suitable for dynamic, large-scale, and privacy-sensitive applications.

The designed data routing strategy for IoT-aided WSN includes the upcoming parts. Traditional data routing works are elucidated in Section 2, and the implementation of a novel routing approach for IoT-aided WSNs with FL is detailed in Section 3. The development of new IRHO and DDQL for the routing process is demonstrated in Section 4. The elucidation of developed ADDQL and FL-based routing and decision-making with multi-objective formulation is given in Section 5. The research validations and the summary of a designed routing system are provided in Section 6 and Section 7.

## 2. Existing Works

### 2.1. Related Works

In 2024, Suresh et al. [23] recommended a federated DRL in IoT-enabled WSNs for routing high-speed data packets. This model employed federated DRL. The suggested framework was employed for dispersing the learning operation across distinct access points or nodes. The simulations were carried out to estimate the efficiency of federated DRL routing. The outcomes illustrated that the model performed more effectively than conventional techniques.

In 2024, Udayaprasad et al. [24] designed an energy-efficient routing approach for large IoT models to improve the scalability of the network and load balancing. This model decreases energy loss with the support of the cluster heads (CHs) in serious conditions that improve energy balance.

In 2023, Arafat et al. [25] implemented an efficient routing protocol. When estimating the CH in each cluster, the residual energy and the node connectivity were concentrated. The routing model was implemented to guarantee the energy-efficient delivery of packets. The results disclosed that the model highly performed well with the conventional routing approaches in distinct functionality measures.

In 2023, Samadi et al. [26] presented a routing mechanism that concentrated on handling the varying topology because of the mobile node’s movement to enhance the lifespan of the network and eliminate energy loss. This mechanism focused on minimizing the control packet’s overhead. The simulation solutions specified the effectiveness of the designed method contrasted to other simulated mechanisms.

In 2023, Prabhu et al. [27] developed a smart routing approach on the basis of DRL. It focused the attributes such as message overhead, sum rate of data, time complexity, and so on for discovering an optimal path for improved functionality in IoT-allowed WSNs. The recommended model was more efficient than other conventional approaches that minimized the node power.

In 2024, Han et al. [28] introduced an enhanced algorithm for discovering the optimal routing approach. The algorithm simulated the ant’s character in the operation of exploring food and optimized the attributes. Through research outcomes, it could be discovered that the introduced approach worked well. These optimization outcomes have positive implications that can enhance smart city construction.

In 2023, Kumar et al. [29] have developed a hybrid algorithm for energy-effective routing in WSNs. This approach was employed to discover the applicable CH in a predefined search space and also discovered the suitable path from primary cluster sensors to CH. The functionality validation illustrated that the suggested approach performed better concerning the lifespan of the network.

In 2024, Bhimshetty et al. [30] explored an energy-efficient routing approach employing RL-based WSNs. This model employed RL for discerning the ideal transmission path from a primary to a sink node. The RL’s training was supported via a reward function that included the data transmission efficacy and energy outflow. The approach was contrasted with other routing approaches. The simulation outcomes illustrate the superiority of the method.

### 2.2. Research Gaps and Challenges

The data routing strategy utilizing federated learning for IoT-enabled WSN represents a complicated method designed to optimize the routing of data packets within dynamic and resource-limited settings [31,32,33]. By enabling multiple IoT devices to design models for better utilization of computational resources across devices, reducing the burden on individual devices and federated learning minimizes the necessity for centralized data collection. Furthermore, this routing strategy is capable of adapting network conditions and user needs, resulting in more efficient routing decisions informed by real-time data. Yet, existing routing models undergo various challenges, which are mentioned in the below points.

- Conventional routing protocols in WSNs frequently result in high energy consumption because of regular data transmission and suboptimal routing paths, which ultimately shortens the network’s lifespan.
- Existing models fail to make effective use of available resources, such as bandwidth and energy, leading to diminished performance and high latency. As the number of IoT devices grows, these existing routing protocols find it challenging to sustain performance and efficiency, resulting in congestion and delays in data transmission.
- In existing models, the computational requirements of deep learning and the communication overhead associated with federated learning still contribute to increased energy consumption in resource-constrained sensor nodes.
- Existing routing models require more frequent updates and data exchanges between devices and the central server, which leads to higher communication costs and energy consumption.
- In existing models, data across different nodes are non-independent and identically distributed, which leads to challenges in training a robust global model. Addressing this issue proposed model requires optimal routing decisions based on energy consumption patterns, leading to more energy-efficient paths and increasing the lifetime of the network.

The features and limitations of existing IoT-based routing in WSN models are provided in Table 1.

## 3. Implementation of Novel Routing Mechanism for IOT-Based WSN with Federated Learning

### 3.1. IoT-Enabled WSN Architecture

The IoT-based WSN network [34] is a dynamic, interconnected, and large SN that combines communication, processing, and sensing. Each SN is an autonomous domain that garners environmental information with constrained communication, processing, and power capabilities. The IoT-allowed WSN is a distributed system where the nodes garner, execute, and send the information to a gateway or edge node. The edge node performs as a middle node among the network infrastructure, including cloud systems and the internet and nodes. The SNs are connected through wireless links, generating a topology with distinct hops. Directly, each node interacts with its nearby nodes, placing within its limits or may relay the information to a sink node with the help of intermediate nodes. Because of the environmental factors, power constraints, and node mobility, the IoT-based WSN approach concentrates on effective data routing and smart decision-making at a network edge. The goal was to reduce the delay, guarantee data integrity and scalability, and optimize energy usage for accommodating and enhancing IoT systems and their related volume of data. Some factors are explained in this architecture to explain the distinct network tasks.

Signal strength: It estimates the strength of a signal obtained among nodes employing the environmental attributes and the distance of a node. It is formulated in Equation (1).

(1) $$Rssi=Q_{j}-\left(Y+10\;m\;\;\log\left(e\right)\right)$$

Here, the attribute Rssi indicates the received signal strength indicator, and the term $Q_{j}$ specifies the transmitted power. The node distance is given as e, and the path loss exponent is specified as m. Finally, the signal attenuation constant is taken as *Y*.

Energy consumption: It validates energy utilized by a specific node at the time. It is formulated in Equation (2).

(2) F=Q×u

Here, the energy and power consumptions are given as F and Q.

Rate of data transmission: It discovers how rapidly data can be sent among the SNs, and it is expressed in Equation (3).

(3) $$S=C\;\;\;\log_{2}\left(1+\frac{\xi }{M_{0}}\right)$$

Here, the noise power and the data transmission rate are given as $M_{o}\;\mathrm{and}\;S$. Then, the “signal-to-interference-plus-noise-ratio” and the available bandwidth are given as ξ and C.

Routing metric: It allows the routing decisions of the network on the basis of link reliability R and the cost *D* with its weighting attributes α and β utilizing the formulation in Equation (4).

(4) SN=αD+βR

Constraint: A constraint of energy *F* forces an energy usage limit of a node to ensure the longevity of a network on the basis of a node’s maximum energy $F_{\max}$. The constraint of bandwidth limits the available bandwidth for high-speed transmission of packets utilizing its maximum rate $S_{\max}$ and rate of data transmission *S*. The constraint of node capacity limits the processing and storage abilities *E* of a specific node and assigns a maximum allowed ability $E_{\max}$. The entire constraints are formulated in Equation (5).

(5) $$F\leq F_{\max};S\leq S_{\max};E\leq E_{\max}$$

**Figure 1:**
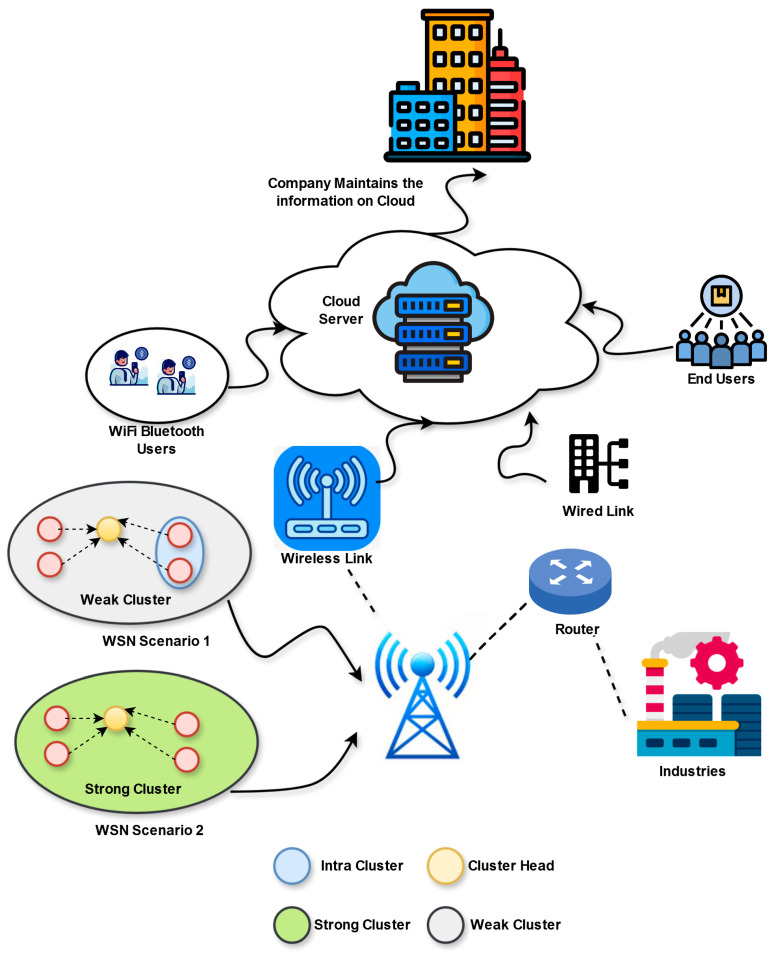
Architectural view of IoT-based WSN for designed routing mechanism.

Other constraints related to routing are on the basis of optimal path selection for available high-speed data transmission. This contains hop constraints and link quality, where the former guarantees the reliability of the optimal link $R\geq R_{\min}$ is attained while a path is handled. The latter handles while sending data through hops $I\leq I_{\max}$ by providing the allowable hops $I_{\max}$ in a path. The architectural diagram of IoT-aided WSNs is provided in Figure 1.

### 3.2. Contributions of FL in IoT-Based WSN

The IoT-aided WSN utilizes interconnected sensors for gathering data from the physical environment and sending it wirelessly. It allows data-driven decision-making as well as automation via the IoT. Nowadays, the concept of FL has been suggested for constructing smart and privacy-improved IoT-aided WSN systems [35]. FL is a machine learning approach where distinct systems, such as IoT sensors, train a model collaboratively without sharing their original data. The integration of FL in IoT-based WSNs allows collaborative and decentralized training without data sharing. It enhances privacy and minimizes expenses. FL secures sensitive data as they are never shared from the sensors to an intermediate server, rectifying a primary concern in the applications of IoT. In the system, the models are locally trained on the edge systems, thus minimizing the dependence on the intermediate server and enhancing the robustness [36]. Moreover, FL can manage distributed, large SNs efficiently. Additionally, FL can enhance the WSN’s scalability by distributing the computational load across distinct SNs. The following advantages are obtained from FL in the IoT-enabled WSN.

- Privacy preservation: FL enables the IoT systems to train the techniques collaboratively without data sharing, which is significant for the applications of privacy sensitivity.
- Data heterogeneity: FL allows the model’s training on distributed and diverse data sources, resulting in highly accurate and robust approaches.
- Reduced bandwidth consumption: By only sending the model updates instead of original data, FL conserves bandwidth and reduces the network traffic, which is relatively significant in the resource-constrained WSN domains.
- Flexibility and scalability: FC can scale to accommodate a huge amount of IoT systems and adapt to varying network conditions, making it effective for distinct applications of WSN.
- Continuous learning: IoT systems can learn and enhance their approaches on the basis of new data continuously, thus enabling the overall network to adapt to varying conditions and environments.
- Enhanced Model Performance: By employing data from a large range of systems, FL can result in highly generalizable and accurate approaches.

The proposed model is innovative in that it is all-accommodating, flexible, and preserves privacy while solving the energy-efficient routing problem in dynamic, resource-constrained wireless sensor networks integrated with other IoT resources through the Internet. The novel approaches, as opposed to the other usual centralized routing or static clustering algorithms that tend to have a narrow capability in adaptability, suffer from high energy depletion, and are difficult to scale, provide a dual-intelligence-based solution combining the distributed learning ability of Federated Learning (FL) with the decision-making force of Adaptive Double Deep Q-Learning (ADDQL). Then, such integration solves the very problem of training routing models in a decentralized manner at every sensor node, therefore ensuring null raw data exchange and protecting privacy and communication costs; these enormous and very highly related subjects are still under-explored concerning real-life IoT applications.

This is another key innovation of the aeration routing policies, which is tied to the design of an ADDQL model endowed with a context-aware and reinforcement-learning-based mechanism, which keeps evolving via continuous interaction with the dynamic WSN environment. Ongoing localized decisions regarding routing for the IoT-based sensor networks, based on the real-time energy status, data traffic, and quality of links for each node, offer solutions to what should be called its own dynamic and heterogeneous characteristics of routing in IoT-based sensor networks today. Another area that has been characterized is hyperparameter tuning of the ADDQL model by the iteration-based random factor of Hippopotamus Optimization (IRHO), indicated now to strengthen the novelty even further. The IRHO is used in the framework of this study to maintain a balanced exploration-exploitation technique through the training process in optimizing convergence speed and route identification accuracy, especially in energy-conserving and dense WSNs. This metaheuristic optimization addition is original in its biological inspiration but also in the unique position it holds in distributed routing-an area where classical optimizers have difficulty in maintaining scalability and timing adaptability in real time.

The most notable aspect of novelty is a real-time dynamic clustering mechanism that forms groups of sensor nodes in clusters: weak clusters and strong clusters. The intelligent distribution of load is put in place so that no node is overloaded, thereby reducing the overall lifetime of the network. This then leads to dynamic clustering based on the operational condition of the nodes, unlike static or probabilistic clustering types. This measures up to improved energy balancing and fault tolerance. In essence, the intelligent routing mechanism is proposed for a relook at intelligent decision-making, along with energy optimization and data privacy functionalities working together in a joint regime: this particularly targets the area of IoT-based WSN applications. By employing the obvious and final combination of FL, RL, and nature-inspired optimization techniques, the current work confines its effort to imaginatively introduce the concept of a new multi-level architecture, which may turn out to be a leap ahead from the state-of-the-art in WSN routing by being an intelligent and efficient solution outmatching the present benchmark solutions, both through theoretical proofs and empirical observations. This novelty aspect carries a strong significance for this work, as it addresses the future of IoT systems, which are having an enormous increase in demand for secure autonomous energy-conscious communication.

The motivation behind the present research is to address the age-old problems that have been energy-efficient and scalable routing in WSNs that fall under the IoT paradigm. As IoT networks turn into huge decentralized systems with billions of interconnected sensor nodes, timely and privacy-preserving data transfer only continues to be more complicated. The conventional routing algorithms would hardly work owing to static settings, centralized decisions, or sheer heuristics: the fundamental dynamic behavior of WSNs would be locked out by such factors detrimental to performance, namely, node mobility, varying traffic patterns, and uneven energy depletion. They are equally oblivious, in one way or the other, regarding some critical modern-day necessities such as data privacy, adaptive learning, and a system-wide scale-out. Empowering individual sensor nodes to make intelligent routing decisions autonomously, safeguard their data privacy, and dynamically adapt to network changes without the use of centralized controllers is the thread running through this paper. Furthermore, the routing in WSNs is an NP-hard problem and thus makes a strong case for designing novel intelligent and distributed mechanisms that would find a tradeoff amongst network parameters of energy consumption, communication delay, overhead in message passing, and throughput for long-term sustainability.

This work integrates Federated Learning (FL) into the RL-based decision-making framework and is perfected with a whole novel iteration-based random factor of the Hippopotamus Optimization (IRHO) parameter-tuning technique. With this kind of approach, sensors can be made to operate in a completely disbursed, privacy-preserving routing mechanism whereby each sensor node independently trains its model using local data and shares only model updates. Whereas previous models rely either on centralized learning models or static heuristics, this approach ensures none of the sensitive environmental data leaves the local node, which is a major leap for privacy-preserving machine learning in WSNs. Embedded within this framework is an Adaptive Double Deep Q-Learning (ADDQL) model, which ensures that nodes are contextually sensitive and can adapt their routing with conditions such as energy depletion of the node, quality of a link, and congestion in traffic data. The provision of hyperparameter adjustment through IRHO offers a totally different optimization level such that models learned under each node converge much better and accurately when compared with conventional techniques such as Particle Swarm Optimization or Genetic Algorithms. The model also provides a new, entirely different clustering function that classifies nodes into weak and strong ones to facilitate intelligent load balancing and pairing of clusters, thereby making data dissemination even more efficient and improving energy utilization. So, in fact, this is a marriage of FL and RL with nature-inspired optimization techniques that make it revolutionary with regard to routing in IoTs-based WSNs, making the solution robustly flexible, scalable, and powerful in countering uncertainties in the topology of the network.

This research has numerous contributions to the field of intelligent IoT routing. First of all, a privacy-preserving decentralized routing architecture is being introduced that uses Federated Learning, making it quite apt for large-scale IoT systems where privacy preservation and data independence are critical issues. Second, the introduction of the ADDQL model presents an algorithm for Deep Reinforcement Learning, allowing continuous learning and adapting to ever-changing WSN conditions such that routing decisions change with the network state in order to be energy-efficient, low-latency, and overhead in messages. Third, an optimizer called IRHO is proposed and appended to calibrate the learning parameters of ADDQL for further convergence and robustness in the decision-making process. Fourth, the clustering technique presented here operates in near real-time while factoring in the energy status of nodes and is thus load-balanced and ensures continuous operation of the network. Finally, the proposed model has also been subjected to extensive simulations and evaluations against contemporary state-of-the-art models to find it potent in its performance on different metrics comprising energy consumption, communication delay, data sum rate, and scalability. This altogether gives a completely intelligent stance to the routing mechanism proposed for the next-generation IoT-WSNs, solving burning issues on the same and marking critical routes for further research on distributed yet secure IoT communications.

### 3.3. Detailed View of Proposed Routing Strategy

**Figure 2:**
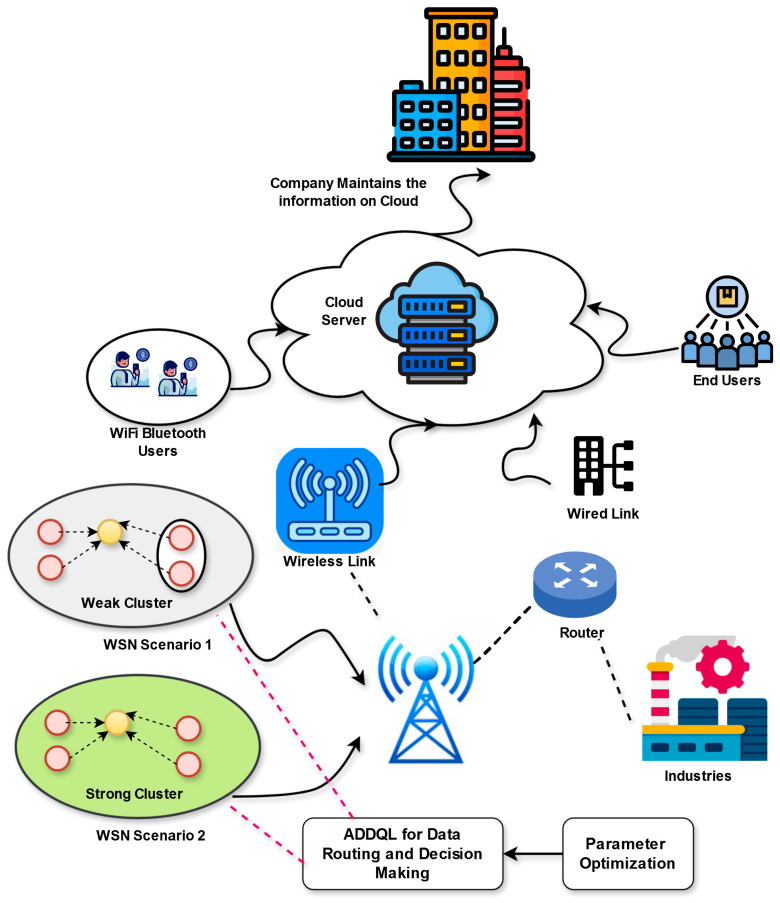
Diagrammatic representation of the designed data routing method using FL-based ADDQL in IoT-based WSN.

The IoT has attained much attention because of its large range of applications including healthcare, smart industry, smart city, transportation, and so on. Often, the IoT links the systems with smart sensing, processing, and communication capabilities. Nevertheless, the IoT is still in the implementation phase concerning its applicability for security-based applications. The IoT-aided WSNs have obtained much attention because of their commercial applications in various sectors. The utilization of swarm intelligence is also largely utilized in IoT, where the amount of intelligent sensing systems are installed in wide regions. The IoT supports handling communication across systems for larger-level transmission. The energy efficiency-aided routing techniques play a significant part. It includes some sensing systems connected to perform a common operation based on the targeted objective. The applications of IoT-aided WSN must offer energy-efficient routing approaches for seamless data transmission because of the limited memory, storage, energy, and computational capabilities of WSN. However, conventional data routing approaches in IoT-based WSNs face some problems, including scalability problems, varying network environments, high energy usage, and so on. The SNs mostly operate on battery nodes, making energy efficiency a serious concern for the longevity of the network. Moreover, the traditional routing models themselves consume specific energy. In addition, the SNs may be deployed or moved in dynamic environments, demanding routing models to adapt to the changing environments. Therefore, this designed framework presents an efficient data routing approach in IoT-aided WSNs. Figure 2 showcases the designed data routing mechanism with the help of FL-based ADDQL in IoT-based WSN.

The suggested approach is inspired to enhance the functionality of IoT-based WSNs by designing a smart data routing mechanism on the basis of FL-aided ADDQL. By employing the abilities of DDQL, the objective is to minimize the complexities of energy efficiency, scalability, relocations, and the network dynamicity related to high-speed packets. The developed framework includes FL for distributing the learning operation across distinct nodes, allowing localized decision-making and improving the routing mechanism’s adaptability. The primary novelty of this work includes combining the federated learning and DDQL models into the model of a smart data routing approach for IoT-based WSNs. The developed model also focuses on the massage overhead, scalability, energy efficiency, communication delay, data sum rate, and time complexity while developing a smart routing. The developed FL-based ADDQL model supports attaining efficient load balancing and improves the network functionality by utilizing double cluster pairing. The developed work presents an FL-aided approach named ADDQL for providing smart routing for high-speed data packets in an IoT-aided WSN. The hyper-parameters in DDQL are optimized utilizing the IRHO, which improves routing efficiency. The primary contribution of this developed model is its effective strategy in focusing on distinct routing factors to an IoT-based WSN routing, improved adaptability to relocations of a node, enhanced scalability, energy efficacy, and the utilization of FL-based ADDQL for distributed decision-making operations in the routing task. Moreover, in the developed framework, the data load is divided into cluster pairs with a weak and strong SN. This division allows processing the load balancing in an ideal IoT-based WSN. Last, the designed framework is utilized to process the learning operation over distinct nodes, and it allows for attaining localized decision-making solutions. The performance validations of this model are performed for this framework over existing approaches. The outcomes portray that the implemented system is effective and provides seamless data transmission.

## 4. Development of a New Iteration-Based Random Factor of HO and Double Deep Q Learning for Routing Process

### 4.1. Developed IRHO

The IRHO is implemented newly in this developed framework.

Purpose: The developed work employs DDQL for performing a smart data routing strategy in IoT-aided WSNs. For obtaining the ideal network performance, the DDQL parameters are optimized during a routing operation. The IRHO is implemented for fine-tuning the developed DDQL’s hyper-parameters. By properly fine-tuning these DDQL parameters, such as back size, number of episodes, and number of steps, data routing becomes energy efficient and also obtains more throughput and PDR. In addition, the delay in the data routing process is relatively minimized.

Reason for choosing HOA: The designed IRHO draws from the inspiration of traditional HOA. The HOA [26] mainly considers the behavior of hippopotamuses. The HOA is a trinary phase approach. By balancing exploitation and exploration, the conventional HOA supports the search operation. The HOA provides relatively effective solutions and helps resolve complex engineering design complexities. There are numerous optimization algorithms invented in the past years for rectifying the challenges of optimization problems. However, a very limited number of algorithms provide satisfactory solutions, and these algorithms also encounter the primary issues of local optima and premature convergence. The conventional HOA properly escapes from these limitations and supports achieving optimal outcomes. Hence, the conventional HOA is taken for this work.

Novelty: The developed system considers the HOA for optimization tasks. Unfortunately, the HOA also has an issue. In the exploitation stage, the HOA leverages a random factor while updating the positions. The random integer is normally chosen between 0 and 1 in the conventional HOA. Most times the conventional HOA takes more time to complete the iteration because of its random integer. This much time utilization may have an impact on the designed data routing process. Therefore, properly handling this issue is necessary. Hence, the IRHO is developed. The IRHO presents a new random factor which is derived on the basis of an iteration integer. This random factor efficiently handles the position-updating process during exploitation. By properly performing the position updating, with the support of this iteration-based random factor, the algorithm becomes powerful and increases the performance of the overall operation. Equation (6) gives the expression of an iteration-based random factor.

(6) $$R_{i}=-I\times \frac{0.02}{Mi}$$

Here, the current and the maximum iterations are specified as I and Mi. In addition, the designed new random factor is indicated as $R_{i}$. By utilizing this random integer, the exploitation phase’s positions are updated and are expressed in Equation (7).

(7) $$K_{b}^{Hippo}:k_{bs}^{Hippo}=k_{bs}+R_{i}.\left(ll_{s}^{local}+\tau 1.\left(uu_{s}^{local}-ll_{s}^{local}\right)\right)$$

Here, the new $b^{th}$ hippopotamus’s position is given as $k_{bs}^{Hippo}$ for $s^{th}$ decision variable. The old $b^{th}$ hippopotamus’s position is given as $k_{bs}$ for $s^{th}$ decision variable. The lower as well as upper regions of the $s^{th}$ search space are provided as $ll_{s}^{local}\;\mathrm{and}\;uu_{s}^{local}$. Moreover, τ1 is a random attribute that is chosen arbitrarily from different scenarios. The modified arbitrary integer is given as $R_{i}$, which is expressed in Equation (6).

**Figure 3:**
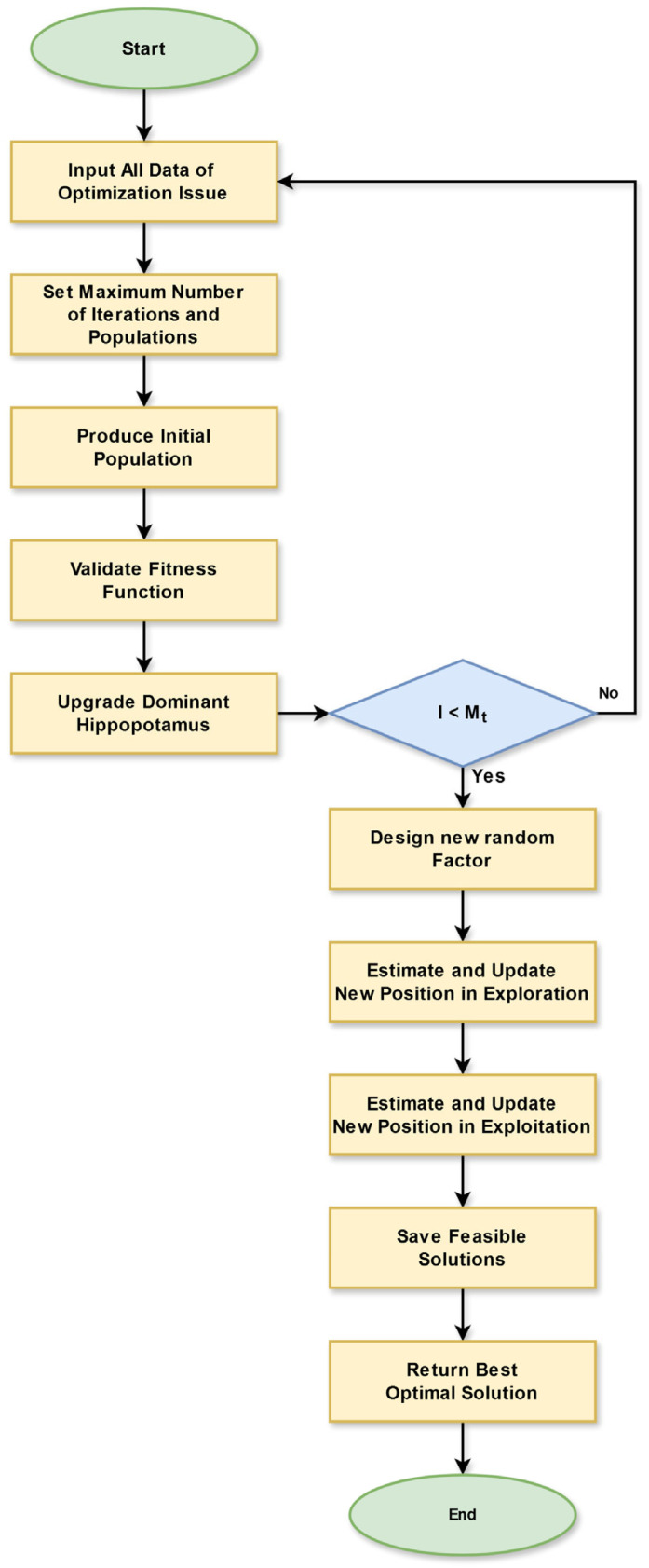
Flowchart of designed IRHO.

Hence, the IRHO is introduced by modifying the exploitation phase of existing HOA with the assistance of a new iteration-based random factor, which helps to improve the performance rate and enhance the parameter tuning in the data routing process. Figure 3 displays the flowchart of the designed IRHO.

### 4.2. Double Deep Q Learning

DDQL [37] is an efficient approach that is highly utilized in IoT-based WSN applications. The concept of double Q learning is developed for minimizing overestimation by dividing the max process in a target into action validation and action selection. Though not entirely decomposed, the target model present in a DQN model offers a natural member for a next value function without the need to present extra models. By incorporating DQN and double Q learning, DDQL is presented. Its update operation is similar to the DQN but exchanges the target $Z^{DQN}$ with $Z^{DDQN}$ as given in Equations (8) and (9).

(8) $$Z^{DQN}=S_{u}+\gamma \;\;\underset{B_{u+1}}{\max}\;\;R\left(T_{u+1},B_{u+1};\theta \right)$$

(9) $$Z^{DDQN}=S_{u}+\gamma R\left(T_{u+1},\;\arg\underset{B_{u+1}}{\max}\;R\left(T_{u+1},B_{u+1};\theta \right);\theta \right)$$

Here, in comparison to the double Q learning, the second network’s weight θ− is exchanged with the target network’s weight θ for the estimation of the present greedy policy. The attribute t∈T indicates the limited state region and the variable Q indicates the evolution behavior. The definite response region is indicated as b∈B that works for an actor. The reward behavior is taken as *S*, and the rebate aspect is defined by γ, where $\gamma \to \left[0,1\right]$ for a defined reward.

The upgrade to the target network remains unchanged from DQN and remains an online network’s periodic replica. This DDQL is maybe the minimal variation to DQN towards the double Q learning. The objective is to obtain the majority of the advantage of double Q learning while keeping the remaining DQN model intact for a better comparison and with less computational overhead.

## 5. Elucidation of Developed Adaptive Double Deep Q-Learning and FL-Based Routing and Decision Making with Multi-Objective Formulation

### 5.1. Introduced ADDQL with FL for Routing and Decision Making

The developed FL-based ADDQL approach is developed for a high-speed routing process in the IoT-based WSN. This process concentrates on utilizing the developed FL-based ADDQL for optimizing the routing decisions. This approach integrates the local decision-making at separate SNs with global coordination for obtaining adaptive and effective routing mechanisms. The developed work mainly utilizes the DDQL approach for routing as well as decision-making. In conventional deep Q learning, the Q-values overestimation can happen because the same neural network is being utilized for both action evaluation and action selection. DDQL mitigates this problem by employing two networks: one for choosing actions and another for estimating them. This supports obtaining highly stable and accurate decisions in WSNs, where accurate data routing is crucial. Moreover, the WSNs are relatively dynamic environments with dynamic topologies and changing traffic patterns. DDQL can adapt to these variations by learning and tuning the routing methods continuously. This is specifically significant in IoT networks, where the environment can quickly vary because of node failures, variable data traffic, and topology changes. However, DDQL demands parameter tuning. Without optimization of DDQL, the agent may take an excessive amount of time to learn the optimal policies or can fail to converge to a better solution within a specific time. To rectify these issues, the DDQL parameters are optimally selected with the support of a newly designed IRHO. The IRHO is specially developed for rectifying optimization problems such as parameter optimization. This algorithm introduces an iteration-aided random integer for improving the exploitation phase thus helping to achieve the optimal solutions in a less amount of time. Thus, ADDQL is designed.

In the majority of IoT-aided environments, sensitive data are produced at nodes, and sharing original data with a central server may raise privacy concerns. In addition, centralized learning techniques can be impractical in large networks, where large amounts of data and a large number of systems make it complex for an intermediate server to handle the entire computations. Therefore, FL is combined with ADDQL. FL-based ADDQL is a powerful strategy for data routing and decision-making in IoT-based WSNs. FL provides a decentralized way of training the models by enabling distinct systems to train collaboratively in a global approach without data sharing. This is highly helpful for IoT-aided WSNs, where energy efficiency, communication overhead, and data privacy are significant factors. Thus, FL-based ADDQL is designed. The primary components of suggested FL-based ADDQL are provided below.

Local decision-making: The routing decisions are made by an individual agent at each IoT node on the basis of local data and policies learned via ADDQL. Initially, each node in the network monitors its local environment continuously. On the basis of the current state, the agent selects an action. ADDQL chooses an action utilizing the online network. Given the present state, the online network estimates the Q-values for entire actions and chooses the action with the greatest Q-value. Once the action is chosen, the node interacts with its environment, resulting in a new state. This interaction also leads to a reward that shows the action state’s success. The primary aspect of ADDQL is learning from the rewards obtained after taking the actions. Equation (10) supports Q-value estimation.

(10) $$Q\left(r,c\right)=Q\left(r,c\right)+\alpha \times \left(x+\gamma \;\max\;Q\left(r^{\prime },c^{\prime }\right)-Q\left(r,c\right)\right)$$

Here, the term $Q\left(r,c\right)$ indicates the Q-value for a pair of state actions $\left(r,c\right)$. The learning rate is considered as *a* and the reward attained from an action in a state is taken as *x*. The maximum Q-value for the subsequent state is taken as $\max\;Q\left(r^{\prime },c^{\prime }\right)$, which is estimated utilizing the target network. The discount factor is specified as γ .

Global coordination: At each node, the local decision is coordinated via the FL model. The improved nodes perform as intermediate coordinators, gathering data from individual SNs and supporting the coordination and training operation. They collect the Q-values and the local policies from involving nodes for upgrading the global routing policy. For the policy aggregation, the global coordination is specified in Equation (11).

(11) $$\theta_{h}=\theta_{h}+\beta \nabla \theta_{m}$$

Here, the local policy parameters are specified as $\theta_{m}$, and the global policy parameters are taken as $\theta_{h}$. The local policy gradients and the aggregation parameter are provided as $\nabla \theta_{m}\;\mathrm{and}\;\beta$.

Rewards: The rewards in the designed FL-based ADDQL are developed for incentivizing reliable and energy efficient routing decisions. The rewards are determined on the basis of measures, including PDR, energy consumption, transmission delay, and other performance measures. Equation (12) provides the reward estimation.

(12) $$x=\alpha_{1}F+\frac{\alpha_{2}}{\delta }+\alpha_{3}E$$

Here, the transmission delay is given as δ, and the energy consumption is taken as *F*. The PDR is specified as *E* and the weighting factors are indicated as $\alpha_{1},\alpha_{2},\;\mathrm{and}\;\alpha_{3}$.

By continuously upgrading the local policies and correlating them via the FL model, the developed system tunes the decisions of routing in the IoT-based WSNs. It allows effective and adaptive routing mechanisms that decrease latency, enhance energy efficiency, and improve network functionality.

Intra-cluster routing: In the developed FL-based ADDQL, the phase of intra-cluster routing concentrates on effective routing within separate clusters. It includes packet forwarding, next hop selection, routing decision, CH selection, and cluster formation.

(i) Cluster formation: In this process, the nodes are categorized into clusters for supporting localized coordination and routing. This operation utilizing K-means clustering is on the basis of distinct criteria, including node capabilities, energy level or proximity. The validation of the distance between the center and the node is specified in Equation (13).

(13) $$d\left(y,p\right)=\sqrt{{\left(v_{y}-v_{p}\right)}^{2}+{\left(r_{y}-r_{p}\right)}^{2}}$$

Here, $y^{th}$ node coordinates are specified as $\left(v_{y},r_{v}\right)$, and the variable $\left(v_{p},r_{p}\right)$ specifies the coordinates of the cluster center $\left(p\right)$. Hence, the SN is allocated to a cluster with a nearby cluster center.

(ii) Selection of *CH*: Within each cluster, the *CH* is chosen to perform as the intermediate coordinating node. The selection operation can focus on attributes such as communication range, residual energy, or other measures for recognizing the highly applicable node as a *CH*. One technique is to select the node with the greatest residual energy as a *CH*. The selection of *CH* is on the basis of residual energy as formulated in Equation (14).

(14) $$CH=\arg\;\max\left(F_{c}\right)$$

Here, $c^{th}$ residual energy is specified as $F_{c}$.

(iii) Routing decision: In this operation, each non-CH node within a cluster utilizes the coordinated FL-based ADDQL approach for making the routing decisions on the basis of learned policies and local observations. The parameters of local policy validate $\theta_{m}$ the available action’s Q-values, assisting the decision-making operation of routing. The selection of routing action on the basis of $\theta_{m}$ is formulated in Equation (15).

(15) $$B_{j}=\arg\;\max\left(Q\left(r_{j},c;\theta_{m}\right)\right)$$

Here, the variable $Q\left(r_{j},c;\theta_{m}\right)$ indicates the Q-value of *c* in on $r_{j}$ the basis of $\theta_{m}$. The variable $B_{j}$ indicates the routing action for *j*.

(iv) Next hop selection: After estimating the routing decisions, each node chooses the subsequent hop on the basis of learned policies and existing nearby nodes within its transmission, such as residual energy, link quality, or the Q-values related to the subsequent hop choices as formulated in Equation (16).

(16) $$Nh_{j}=\arg\;\max\left(Q\left(r_{j},c;\theta_{m}\right)\right)$$

(v) Packet forwarding: Once the subsequent hop is chosen, a node sends the packet to a selected neighbor, allowing it to move the cluster to its destination. The operation of packet forwarding continuously starts until the packet meets the sink or CH, based on a network framework and it is formulated in Equation (17).

(17) $$Forward\left(packet,Nh_{j}\right)$$

This specifies sending a packet to a chosen subsequent hop $Nh_{j}$ for then routing in a cluster.

Inter-cluster routing: This stage concentrates on effective routing decisions and data transmission among distinct clusters in IoT-aided WSNs. This state includes packet forwarding, inter-cluster communication, and path selection.

(i) Inter-cluster communication: It allows the replacement of data and information among CHs or specific nodes specifying each cluster. It supports the cooperation and coordination for effective routing decisions and transmission of inter-cluster data. The path selection is expressed in Equation (18).

(18) $$W=\arg\;\max\left(Q\left(r,c;\theta_{h}\right)\right)$$

Here, $c^{\prime }s$ Q-value in *r* on the basis of $\theta_{h}$ is indicated as $Q\left(r,c;\theta_{h}\right)$. The path with the greatest Q-value is chosen as an ideal path for the communication of inter-cluster.

**Figure 4:**
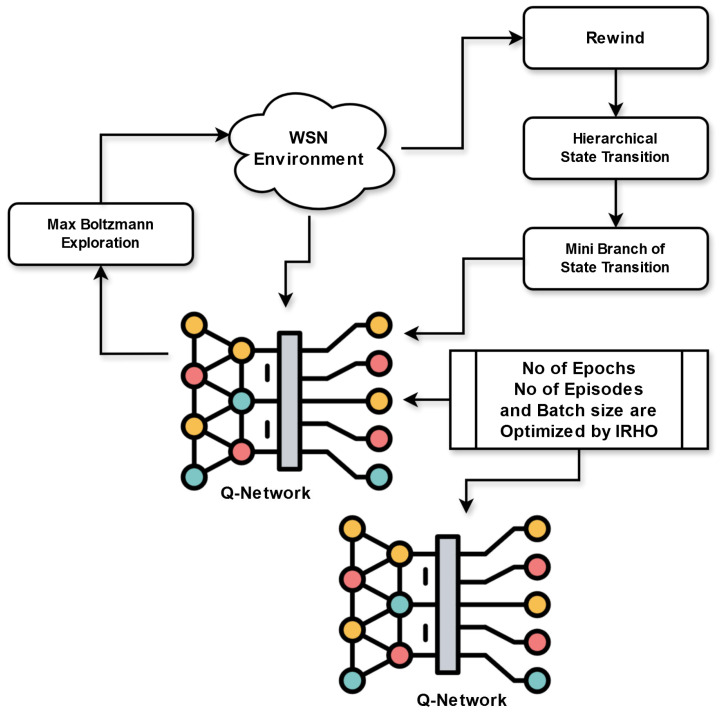
Effective data routing and decision-making operations in the IoT-aided WSN utilizing the developed FL-based ADDQL.

The stages, such as route selection, packet forwarding, and a next hop selection, are the same as in intra-cluster routing.

(ii) Inter-cluster data transmission: The packet forwarding is performed until it meets the final cluster, where it is obtained by a specific node or destination CH. The inter-cluster data transmission operation allows interaction among distinct clusters in a WSN. The developed FL-based ADDQL approach utilizes decision-making and coordinated learning operations for optimizing the inter-cluster routing operation. By utilizing the FL model, the Q-values and the local policies involving SNs are gathered and coordinated for enhancing the total routing functionality and resource usage in the communication of the inter-cluster. Figure 4 shows data routing and decision-making operations in the IoT-aided WSN utilizing developed FL-based ADDQL.

### 5.2. Multi-Objective Formulation

The designed ADDQL utilizes the IRHO for fine tuning the parameters of DDQL. The fine-tuning of DDQL’s parameters, including the number of steps, number of episodes, and batch size is important for improving the overall network functionality and also making an effective decision-making process. The objective function of this operation is shown in Equation (19).

(19) $$Ob=\underset{\left\{N^{s},N^{e},B^{z}\right\}}{\arg\;\min}\left[\frac{1}{Tp}+\frac{1}{Pdr}+Ec+De\right]$$

Here, the optimized number of steps in DDQL is considered as $N^{s}$ and is chosen among [1000–10000]. The optimized number of episodes in DDQL is considered as $N^{e}$ and is chosen among [10000–100000]. The optimized batch size in DDQL is considered as $B^{z}$ and is chosen among [4–128]. In this process, the throughput and PDR are maximized, and also the energy consumption and the latency are minimized.

Throughput: It defines the rate at which data are successfully transmitted via the network. It is expressed in Equation (20).

(20) $$Tp=\frac{T_{pack}}{T_{time}}$$

Here, the successfully transmitted total number of packets and the overall time utilized for delivering the packets are indicated as $T_{pack}\;\mathrm{and}\;T_{time}$.

PDR: It is the ratio of the packet’s number $T_{deli}$ delivered successfully to a destination to an overall packet number $T_{pase}$ transmitted by the source node. It is formulated in Equation (21).

(21) $$Pdr=\frac{T_{deli}}{T_{pase}}$$

Energy consumption: It defines the overall amount of energy utilized by SNs during the transmission of data, reception, and other tasks. It is expressed in Equation (22).

(22) $$Ec=E_{t}+E_{r}+E_{i}$$

Here, the transmission energy, reception energy, and the idle energy are given as $E_{t},E_{r},\;\mathrm{and}\;E_{i}$.

Delay: It defines the time it utilizes for a packet to transmit from a source to destination nodes. It is formulated in Equation (23).

(23) $$De=D_{t}+D_{p}+D_{q}+D_{r}$$

Here, the “transmission delay, propagation delay, queuing delay, and processing delay” are indicated as $D_{t},D_{p},D_{q},\;\mathrm{and}\;D_{r}$.

**Figure 5:**
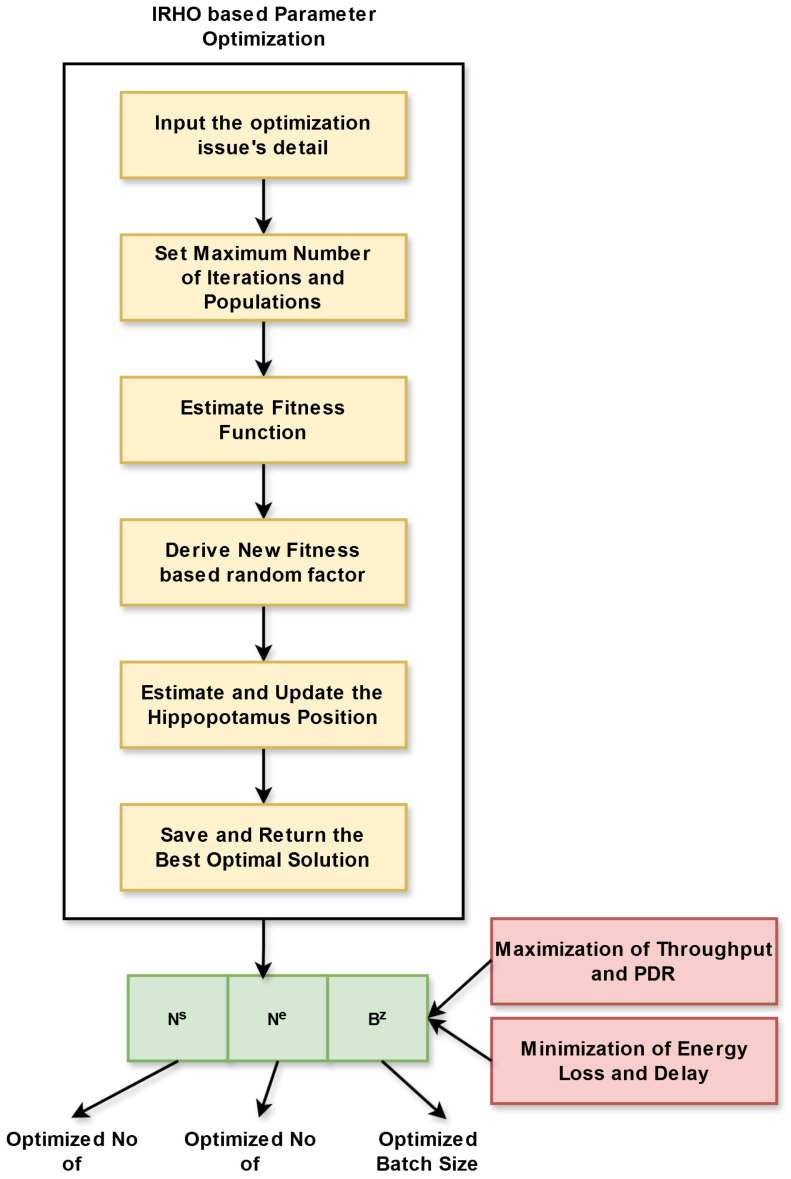
Solution encoding diagram of parameter tuning.

Thus, the IRHO helps to fine tune the parameters of DDQL and helps to achieve optimal performance in IoT-enabled WSN. Figure 5 displays the solution encoding diagram for parameter tuning.

The advanced additive double deep Q-learning with iteration-based random objective hippopotamus optimization (ADDQL-IRHO) approach is based on core design strategies for addressing the challenges of IoT-based WSN environments. First, the amalgamation of Federated Learning into ADDQL provides intelligent decentralized learning among sensor nodes, thus cutting off the excessive communication that would have been incurred by having a central data collection process. This design allows optimized intelligent decisions to be made in deciding a real-time route based on learned experiences and localized observations rather than having to go for the centralized collection of data. Such autonomy, as opposed to centralized methods for routing, enhances dynamic adaptability to changing topologies and variable data loads.

The use of this nice factor of IRHO has led to tuning important parameters of the ADDQL model for faster convergence and higher preciseness in routing. This algorithm is based on the intelligent behavior of hippopotamuses in dynamic environments. Therefore, it simulates the well-explored-exploit balance in the learning model. Consequently, ADDQL-IRHO can identify the best routing paths using less energy and more yield and quickly under high traffic or mobility conditions. Furthermore, the dynamic clustering of nodes into weak and strong pairs in the model provides a better load-balancing scheme that minimizes the stress level on energy-constrained nodes and will last the lifetime of the entire network.

Apart from these aforementioned factors, the strength imparted to the model against scalability and the growing density of networks is one of the other major distinguishing performance factors. Where old architectures die with ever-increasing latency and message collision with growing numbers of nodes, the new approach, which is based on FL, survives and thrives through efficient, consolidated routing alone across all nodes. This has also been further confirmed with experiments that show that the proposed method outperforms existing algorithms on various criteria, such as reduced temporal complexity, minimized message overhead, and a considerably improved data sum rate. These bright spots point to the ability of the proposed method as potentially scalable, energy aware, and intelligent for modern applications in the IoT-based wireless sensor networks.

## 6. Results and Discussion

### 6.1. Simulation Setup

An effective data routing mechanism in IoT-aided WSN was executed by the MATLAB 2020a platform. The designed system leveraged the IRHO, which utilized “10 populations, 100 maximum iterations, and 3 chromosome lengths”. The traditional algorithms as well as methods such as Gazelle Optimization Algorithm (GOA) [38], Arithmetic Optimization Algorithm (AOA) [39], Yellow Saddle Goatfish Algorithm (YSGA) [40], HOA [26], FDRL [1], SDN [2], DRL [5], and DDQL [38] were utilized for examining the designed framework’s performance. The developed work utilized state values such as 15, 30, 45, 60, and 75 for validation. The state defines the present condition or situation of the environment that the agent is in.

### 6.2. Performance Measures

The following measures are utilized for examining the efficacy of the designed data routing strategy.

The measures such as throughput, PDR, energy consumption, and delay are explained in Section 5.2.

Message overhead: It defines the number of extra control messages required to deliver data packets successfully, contrasted to the number of original measures being transmitted.

Sum rate: It defines the overall achievable data rate of entire links in a network, which is estimated by adding an individual link’s data rate.

Time complexity: It estimates the efficacy of a routing approach by validating how the runtime varies as the input data size increases.

Accuracy: It defines how closely the optimal path matches the original path for timely data transmission.

### 6.3. Convergence Analysis

**Figure 6:**
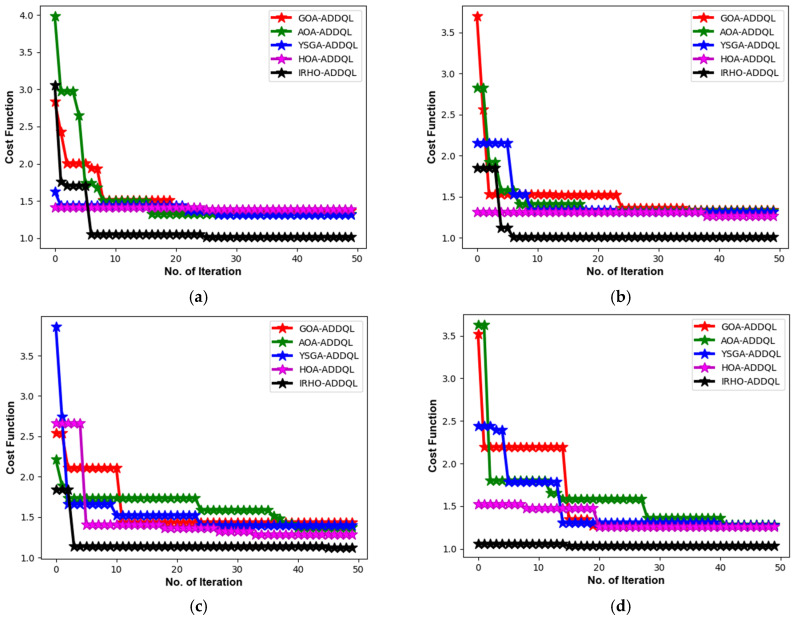
Convergence investigation of the designed IRHO-ADDQL over conventional algorithms based on different numbers of nodes such as (**a**) 50, (**b**) 100, (**c**) 150, and (**d**) 200.

Figure 6 showcases the designed IRHO-ADDQL’s convergence estimation over existing algorithms. For a distinct number of nodes, the designed IRHO’s functionality in ADDQL-aided data routing and decision-making operations is estimated. At the 20th iteration in a 50th node value, the designed IRHO-ADDQL’s cost function is decreased to 50% of GOA-ADDQL, 45% of AOA-ADDQL, 46% of YSGA-ADDQL, and 47% of HOA-ADDQL, respectively. The decreased cost function rates in all node values portray that the implemented IRHO provides optimal and promising solutions for the ADDQL model than the conventional algorithms. In addition, the enhanced convergence rate of IRHO-ADDQL ensures that the algorithm can choose optimal solutions quickly, even in complex scenarios.

Convergence analysis was conducted based on different node abundances, i.e., 50, 100, 150, and 200, thus illustrating how well and efficiently the proposed method minimizes the cost function with different performing iterations across mixed scales of wireless sensor networks (WSNs). During some instance-in-point, for example, in the 20th iteration for a 50-node network, about a 50% reduction in the cost function is shown by the IRHO-ADDQL model in comparison to GOA-ADDQL, 45 % compared to AOA-ADDQL, 46% against YSGA-ADDQL, and 47% against HOA-ADDQL. These reductions show that IRHO is vastly superior in directing the ADDQL framework to energy-efficient and optimal routing solutions in the early stages of convergence, which is significant in dynamic IoT settings because of the scarcity of computational and energy resources and an emphasis on fast decisions.

The cost function in this context incorporates a composite performance index consisting of energy consumption, routing delay, node load balancing, and communication overhead, and a lower cost means the routing decisions are better optimized in these key attributes. The algorithmic inner working of the IRHO algorithm introduces adaptive randomness that responds to the feedback given by the iteration in order to diversify the search space during the initial learning phases and focus the search near the optimal area during the later stages, thus balancing exploration and exploitation well. A departure from traditional optimizers, which tend to become trapped in local minima in high-dimensional dynamic optimization problems, IRHO is designed for steady and aggressive descent over the cost function. It also exhibits stable convergence across different network scenarios, as opposed to working under definite circumstances alone, thus providing upward support for its scalability and applicability in deploying both small-scale and large-scale scenarios of IoT-based WSN.

### 6.4. Statistical Analysis

Table 2 illustrates the statistical examination of implemented IRHO-ADDQL over other algorithms. This examination leverages the statistical factors for analyzing IRHO’s functionalities in the designed ADDQL. Here also, distinct node values are selected and showcase that the designed IRHO-ADDQL shows stable and reliable performance in all number of node values. At the best factor in the 100th node value, the designed IRHO-ADDQL’s performance is increased by 33% of GOA-ADDQL, 32% of AOA-ADDQL, 30% of YSGA-ADDQL, and 26% of HOA-ADDQL, respectively. Therefore, it is guaranteed that the IRHO is relatively effective in fine-tuning DDQL parameters and also helps in enhancing the network throughput and PDR rates more highly than the other conventional algorithms. So, the data routing process in the IoT-enabled WSN becomes efficient.

### 6.5. Reward Analysis

**Figure 7:**
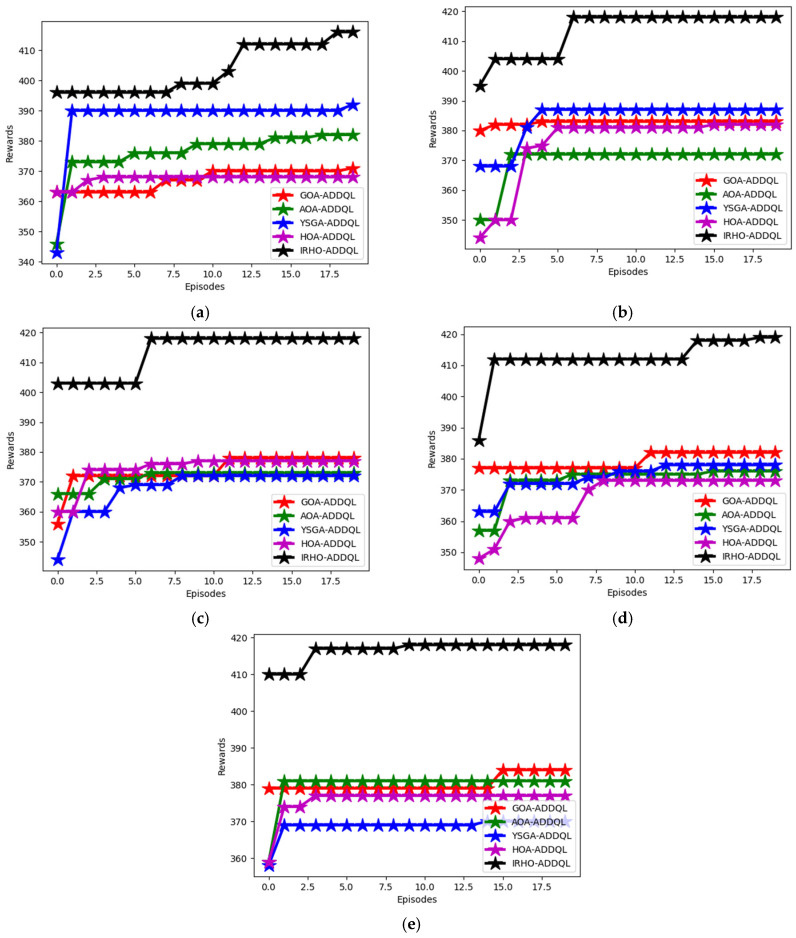
Algorithms-based analysis: Designed IRHO-ADDQL’s reward analysis over traditional algorithms and methods based on distinct state values such as (**a**) 15, (**b**) 30, (**c**) 45, (**d**) 60, and (**e**) 75.

**Figure 8:**
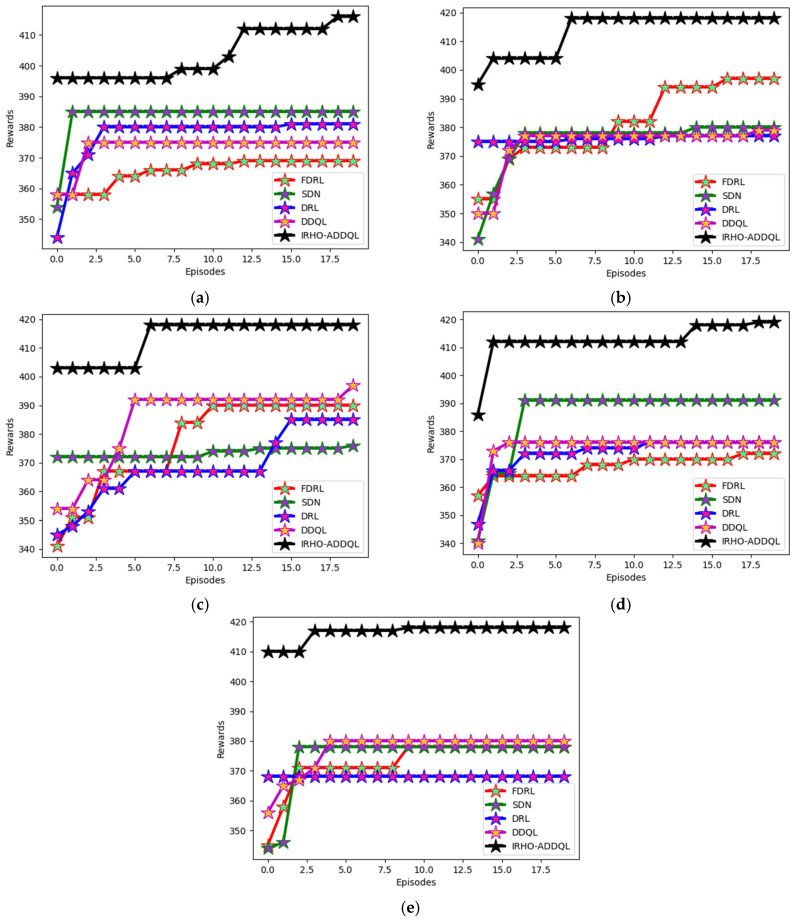
Model-based analysis: Designed IRHO-ADDQL’s reward analysis over traditional algorithms and methods based on distinct state values such as (**a**) 15, (**b**) 30, (**c**) 45, (**d**) 60, and (**e**) 75.

The developed IRHO-ADDQL’s reward analysis is performed in Figure 7 and Figure 8 over conventional algorithms and the methods utilizing distinct state values. The developed model includes DDQL, which is a reward-penalty-aided approach. By varying state values, reward evaluation is performed for this designed IRHO-ADDQL. At the 10th episode value in the 60th state, the suggested IRHO-ADDQL’s reward is enhanced by 10.46% of FDRL, 5.1% of SDN, 9.97% of DRL, and 8.75% of DDQL, respectively. The experimental validations portray that the implemented IRHO-ADDQL network achieves relatively higher rewards than the conventional approaches and ensures seamless data transmission in the IoT-based WSN. In addition, by obtaining high rewards, the developed IRHO-ADDQL provides accurate decisions in the routing process.

The comparative analysis of reward of the proposed IRHO-ADDQL model from many of the traditional and state-of-the-art reinforcement learning-based methods, namely FDRL (Federated Deep Reinforcement Learning), SDN (Software Defined Networking-based RL), DRL (Deep Reinforcement Learning), and DDQL (Double Deep Q-Learning), under different state conditions with specific state values of 15, 30, 45, 60, and 75 are presented in Figure 7. This research specifically is very critical in judging how effectively the framework of IRHO-ADDQL ends up learning the optimal routing policies maximizing the cumulative reward over multiple episodes and, thus, for routing in WSNs based on IoTs, means much cleverer or smarter decisions associated with improving energy efficiency, latency, and reliability in packet delivery. The reward system of this framework follows the dynamic reward-penalty structure: positive routing (such as low-latency or low-energy paths) is rewarded; poor performance (such as route loops or high energy-consuming paths) is penalized. Quite interestingly, at an important benchmark, the 10th episode, at the 60th state, in the case of IRHO-ADDQL, it outperformed all other methods, i.e., FDRL by 10.46%, SDN by 5.1%, DRL by 9.97%, and even DDQL itself by 8.75%.

This difference in performance levels reflects how the iteration-based random factor Hippopotamus Optimization method has been brought into the standard DDQL framework, as the optimizer efficiently manages to equip action-value updates and policy selections in an intelligent yet decentralized way. Improving the reward at all state values indicates that the model is well-protected and very flexible with respect to continuously and dynamically changing environments because conditions and states frequently differ in WSNs. High reward acquisition not only indicates that the model predicts the most efficient routes under fluctuating conditions of network dynamics but also ensures fast convergence of the learning process into optimal policies with better generalization over unseen states. So, the IRHO-ADDQL emerges even superior to being on a numerical scale as it succeeds in showing consistently high-quality decision-making processes that significantly enhance the standard of real-time, distributed routing in IoT-based WSN frameworks.

### 6.6. Penalty Analysis

**Figure 9:**
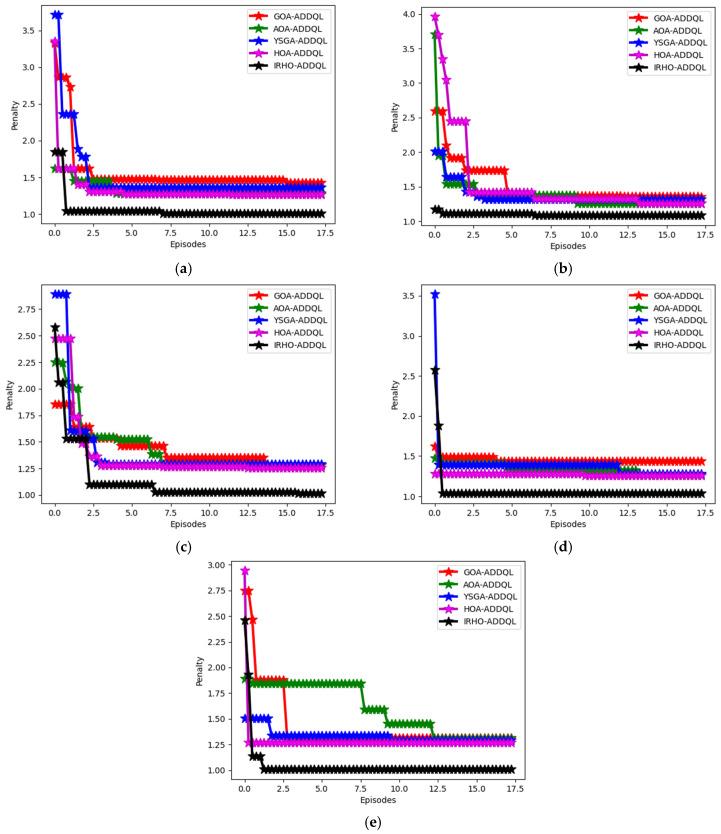
Algorithm-based analysis: Designed IRHO-ADDQL’s penalty analysis over traditional algorithms and methods based on distinct state values such as (**a**) 15, (**b**) 30, (**c**) 45, (**d**) 60, and (**e**) 75.

**Figure 10:**
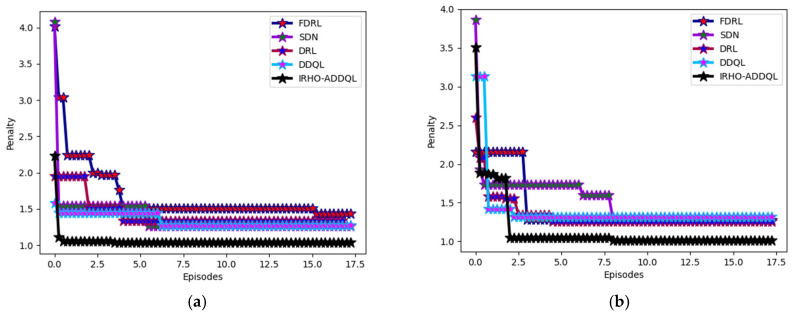

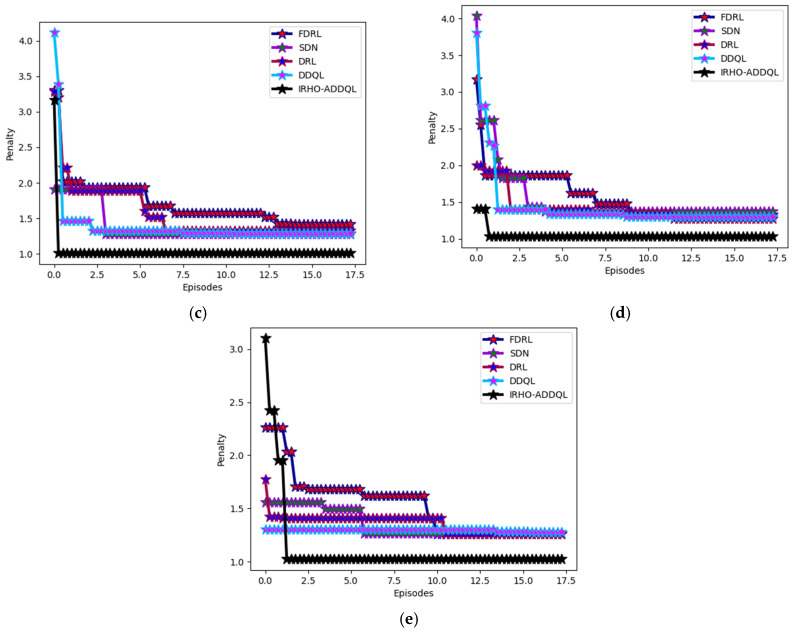
Model-based analysis: Designed IRHO-ADDQL’s penalty analysis over traditional algorithms and methods based on distinct state values such as (**a**) 15, (**b**) 30, (**c**) 45, (**d**) 60, and (**e**) 75.

The implemented IRHO-ADDQL’s penalty analysis is shown in Figure 9 and Figure 10 over traditional heuristic approaches and the methods utilizing distinct state values. This experiment also considers the distinct state values for analyzing the rewards of the suggested IRHO-ADDQL. At the 15th episode in the 75th state, the designed IRHO-ADDQL’s penalty is minimized by 58.75% of GOA-ADDQL, 59.37% of AOA-ADDQL, 59.12% of YSGA-ADDQL, and 56.25% of HOA-ADDQL, respectively. The minimized penalty values of the developed IRHO-ADDQL ensure that the routing and decision-making operations in the IoT-enabled WSN become more accurate and robust than the other techniques. Moreover, the minimized penalty values guarantee the IoT-based WSN network becomes reliable and optimal for transmitting data packets seamlessly and rapidly.

### 6.7. Performance Analysis

**Figure 11:**
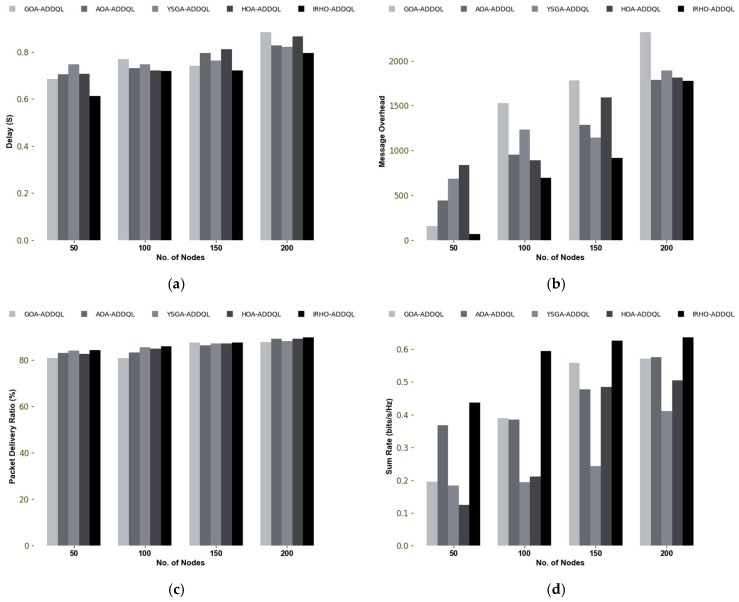

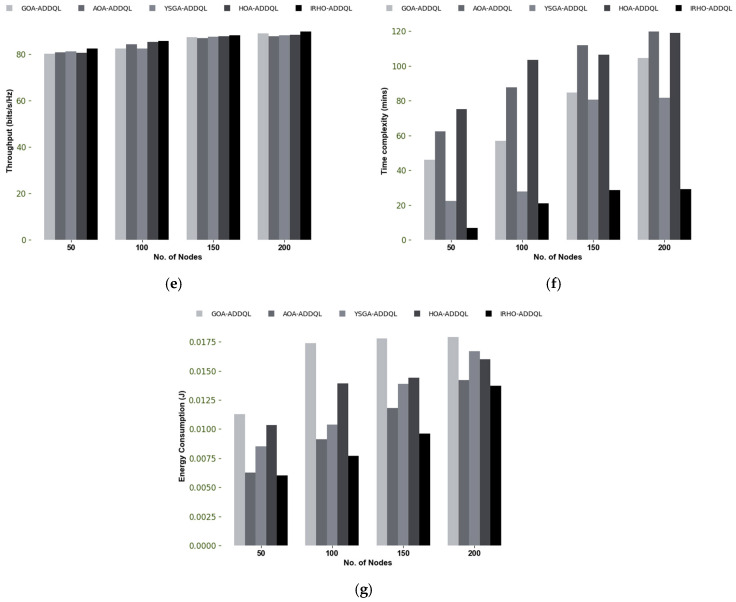
Performance investigation of IRHO-ADDQL-aided data routing mechanism over conventional algorithms in terms of (**a**) Delay, (**b**) Message overhead, (**c**) PDR, (**d**) Sum rate, (**e**) Throughput, (**f**) Time complexity, and (**g**) Energy consumption.

Figure 11 showcases the performance investigation of the designed IRHO-ADDQL-aided data routing mechanism in IoT-based WSNs. The designed approach utilizes FL and ADDQL for performing the routing and decision-making operations. When the node value is 200, the designed IRHO-ADDQL’s throughput is enhanced by 4.7% of GOA-ADDQL, 5.88% of AOA-ADDQL, 5.64% of YSGA-ADDQL, and 5.41% of HOA-ADDQL, respectively. This examination also showcases that the suggested IRHO-ADDQL provides lower delays, message overhead, time complexity, and energy consumption than the traditional algorithms. On the other hand, the designed IRHO-ADDQL achieves a higher PDR and sum rate than the existing algorithms. Therefore, the designed IRHO-ADDQL is a highly promising approach for data routing than the other algorithms in IoT-based WSN.

### 6.8. Training Accuracy Analysis

**Figure 12:**
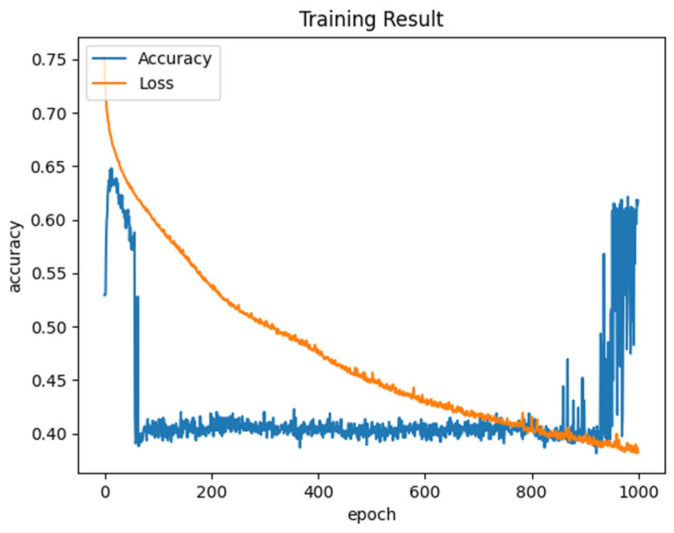
Training accuracy analysis of designed IRHO-ADDQL.

Figure 12 illustrates the training accuracy validation utilizing epoch values. In this investigation, the loss and accuracy of the training process are analyzed. When the epoch value increases, the designed IRHO-ADDQL’s training accuracy is enhanced, while the designed IRHO-ADDQL’s training loss is reduced drastically. Hence, it is proved that the suggested IRHO-ADDQL approach in the data routing process is highly efficient and provides optimal performance for the IoT-based WSN applications than the conventional approaches.

## 7. Conclusions

This research study has presented an intelligent data routing system for IoT-based WSNs based on FL-aided ADDQL. This work primarily focused on issues such as limited energy efficiency, scalability, node relocations, network dynamicity, and so on. This work introduced an FL-aided approach named ADDQL for providing optimal routing for the data packets in IoT-based WSNs. Here, the IRHO was supported for optimizing the hyper-parameters of DDQL, which improved the routing efficiency. The developed system provided better routing and distributed decision-making solutions in varying scenarios. In the designed approach, the instant data load is partitioned into cluster pairs with weak as well as strong SNs. ADDQL was utilized for performing the learning task over distinct nodes, and it enabled the attainment of localized decision-making solutions. The performance has been investigated for this approach over traditional works. The PDR of the developed IRHO-ADDQL increased by 5.74% of GOA-ADDQL, 3.44% of AOA-ADDQL, 4.59% of YSGA-ADDQL, and 4.02% of HOA-ADDQL, respectively, at the 200th node value. The experiment ensured that the designed smart data routing system for IoT-based WSN minimized the delay, time, and message overhead and maximized the throughput and PDR. Thus, the developed framework provided an effective mechanism for routing in IoT-based WSN and enhanced the scalability and energy efficiency. However, the developed framework was not applied in real-time IoT-based WSN applications, which possess complexities for routing approaches. In future work, the developed framework will be applied to real-time applications to showcase its effectiveness. Moreover, the developed system will be extended by incorporating robust security protocols and encryption approaches for protecting sensitive data during transmission.

## Figures and Tables

**Table 1:** Features and Challenges of Existing IOT-Based Routing In WSN.

Author	Methodology	Features	Challenges
Suresh et al. [23]	FDRL	This method allows individual nodes to retain their data effectively.	Communication delays are high in this method.
Udayaprasad et al. [24]	SDN	This model allocates the resources dynamically, which enhances the security of data transmission.	It introduces latency and performance overhead, particularly in large-scale networks.
Arafat et al. [25]	DECR	This method increases the overall lifetime of a network by communicating with nearby CH rather than transmitting data over long distances.	This method enhances the complexity of the system and the overhead.
Samadi et al. [26]	IERMIoT	It manages dynamic changes in network topology, which provides reliable communication among the network.	As the number of systems in an IoT network enhances, maintaining efficient routing becomes more complex in this method.
Prabhu et al. [27]	DRL	DRL models enhance their decision-making accuracy by optimizing their policies through comprehensive training.	DRL models often require substantial computational power.
Han et al. [28]	IACA	By optimizing routing paths according to energy consumption, the IACA model prolongs the lifespan of sensor nodes and enhances the overall efficacy of the network.	IACA may become stuck in local optima, resulting in suboptimal routing solutions.
Kumar et al. [29]	WAOA	WAOA significantly lowers energy consumption by optimizing the choice of cluster heads and routing paths	The computational requirements of the hybrid algorithm might affect the capabilities of certain nodes.
Bhimshetty et al. [30]	FCM	This model enhanced the overall network performance, resulting in improved data delivery rates and minimized delay.	As the number of systems within an IoT network grows, the complexity of the RL model may also rise, leading to scalability issues.

**Table 2:** Statistical Examination of Implemented IRHO-ADDQL over Traditional Algorithms Using Different Node Values.

Terms	GOA-ADDQL [30]	AOA-ADDQL [31]	YSGA-ADDQL [32]	HOA-ADDQL [26]	IRHO-ADDQL
**Number of nodes:50**					
**Best**	1.367221717	1.327008471	1.315670531	1.386346225	1.011547396
**Worst**	2.831332739	3.980690416	1.626239545	1.413434657	3.05679448
**Mean**	1.525548043	1.556621933	1.377247497	1.399890441	1.138022068
**Median**	1.367369675	1.327008471	1.365544027	1.399890441	1.031302392
**Standard deviation**	0.301230716	0.547754415	0.066549634	0.013544216	0.343626462
**Number of nodes:100**					
**Best**	1.332472208	1.326900929	1.309793984	1.263856025	1.005139131
**Worst**	3.693216301	2.827998823	2.15336773	1.308682711	1.848050982
**Mean**	1.493420211	1.443468693	1.431838731	1.298085009	1.077143893
**Median**	1.36076299	1.326900929	1.309793984	1.308682711	1.005139131
**Standard deviation**	0.362508366	0.309654231	0.271264286	0.018859319	0.228424785
**Number of nodes:150**					
**Best**	1.431118549	1.37264242	1.400124174	1.28150209	1.119324721
**Worst**	2.53964408	2.211246869	3.855050805	2.658863984	1.840534949
**Mean**	1.596759987	1.6173011	1.552274902	1.472464296	1.175925251
**Median**	1.431118549	1.583170264	1.400124174	1.366782707	1.135191288
**Standard deviation**	0.321650977	0.171043547	0.38560915	0.39866155	0.167977677
**Number of nodes:250**					
**Best**	1.270923584	1.279334609	1.274006028	1.258950045	1.032041414
**Worst**	3.520591482	3.625904359	2.436391899	1.517943624	1.057422979
**Mean**	1.580626418	1.597013528	1.498258271	1.351401436	1.039655884
**Median**	1.270923584	1.579958457	1.30830824	1.258950045	1.032041414
**Standard deviation**	0.49503774	0.454303056	0.358325157	0.11414003	0.011631294

## Data Availability

Data are contained within the article.
